# Predicting 10-year stroke mortality: development and validation of a nomogram

**DOI:** 10.1007/s13760-021-01752-9

**Published:** 2021-08-18

**Authors:** Weronika A. Szlachetka, Tiberiu A. Pana, Mamas A. Mamas, Joao H. Bettencourt-Silva, Anthony K. Metcalf, John F. Potter, David J. McLernon, Phyo K. Myint

**Affiliations:** 1 Ageing Clinical and Experimental Research Team, Institute of Applied Health Sciences, School of Medicine, Medical Sciences and Nutrition, University of Aberdeen, Aberdeen, UK; 2 Keele Cardiovascular Research Group, Centre for Prognosis Research, Keele University, Stoke-on-Trent, UK; 3 Norwich Medical School, University of East Anglia, Norwich, UK; 4 Clinical Informatics, Department of Medicine, University of Cambridge, Cambridge, UK; 5 Norfolk and Norwich University Hospital, Norwich, UK; 6 Norwich Cardiovascular Research Group, Norwich Medical School, University of East Anglia, Norwich Research Park, Norwich, UK; 7 Medical Statistics Team, Institute of Applied Health Sciences, School of Medicine, Medical Sciences and Nutrition, University of Aberdeen, Aberdeen, UK; 8 Aberdeen Royal Infirmary, NHS Grampian, Aberdeen, UK; 9 School of Medicine, Medical Sciences and Nutrition, Polwarth Building, Room 4:013 Foresterhill, , Aberdeen, AB25 2ZD Scotland UK

**Keywords:** Ischaemic stroke, Long-term mortality, Prediction score, Prognosis, Cerebrovascular disease

## Abstract

Predicting long-term stroke mortality is a clinically important and unmet need. We aimed to develop and internally validate a 10-year ischaemic stroke mortality prediction score. In this UK cohort study, 10,366 patients with first-ever ischaemic stroke between January 2003 and December 2016 were followed up for a median (interquartile range) of 5.47 (2.96–9.15) years. A Cox proportional-hazards model was used to predict 10-year post-admission mortality. The predictors associated with 10-year mortality included age, sex, Oxfordshire Community Stroke Project classification, estimated glomerular filtration rate (eGFR), pre-stroke modified Rankin Score, admission haemoglobin, sodium, white blood cell count and comorbidities (atrial fibrillation, coronary heart disease, heart failure, cancer, hypertension, chronic obstructive pulmonary disease, liver disease and peripheral vascular disease). The model was internally validated using bootstrap resampling to assess optimism in discrimination and calibration. A nomogram was created to facilitate application of the score at the point of care. Mean age (SD) was 78.5 ± 10.9 years, 52% female. Most strokes were partial anterior circulation syndromes (38%). 10-year mortality predictors were: total anterior circulation stroke (hazard ratio, 95% confidence intervals) (2.87, 2.62–3.14), eGFR < 15 (1.97, 1.55–2.52), 1-year increment in age (1.04, 1.04–1.05), liver disease (1.50, 1.20–1.87), peripheral vascular disease (1.39, 1.23–1.57), cancers (1.37, 1.27–1.47), heart failure (1.24, 1.15–1.34), 1-point increment in pre-stroke mRS (1.20, 1.17–1.22), atrial fibrillation (1.17, 1.10–1.24), coronary heart disease (1.09, 1.02–1.16), chronic obstructive pulmonary disease (1.13, 1.03–1.25) and hypertension (0.77, 0.72–0.82). Upon internal validation, the optimism-adjusted c-statistic was 0.76 and calibration slope was 0.98. Our 10-year mortality model uses routinely collected point-of-care information. It is the first 10-year mortality score in stroke. While the model was internally validated, further external validation is also warranted.

**Supplementary Information:**

The online version contains supplementary material available at 10.1007/s13760-021-01752-9.

## Introduction

Despite prevention, treatment advances and extensive research, stroke continues to pose a significant global health burden. It causes significant societal and economic hardship, with stroke costs representing 3–4% of total health care expenditures in Western countries [1]. It is the second leading cause of death worldwide [2]. Similarly, in the UK, cerebrovascular accidents are the third commonest cause of death [3].

Clinical prediction scores provide clinicians, patients and their families with information that can facilitate decisions about their care, identifying those at high risk who require immediate intervention. They also provide very important information on patient prognosis. Numerous attempts have been made to create a reliable, clinically useful and easy-to-use stroke prediction score. Outcomes considered in previously developed scores include in-hospital, 3 months, 6 months, 1-year mortality, stroke recurrence as well as functional outcomes: mRS (modified Rankin Score) and hospital length of stay [4–6]. None of the existing scores is widely used in clinical practice. Some of the potential reasons for that can be a very complex scoring system, included variables that are not routinely collected, or imaging results required, that may not be available in some settings. In recent years, life expectancy of stroke survivors has been increasing—from 1983 to 1994, it increased by 22.9% for men and 12.9% for women [7]. Therefore, a prediction model for mortality in the longer term (10 years) is needed to guide long-term prognosis and can be used as prognosis tool to stratify patients participating in clinical studies. Whilst many stroke scores provide clinicians with short-term mortality and disability prognosis, so far none of them can predict 10-year mortality.

In the current study, we aimed to develop and internally validate a score using readily available clinical information at the time of stroke to predict 10-year ischaemic stroke mortality.

## Methods

### Data collection and participants

In this prospective cohort study, we included participants from the Norfolk and Norwich Stroke and TIA Register (NNSTR) [8] database which includes consecutive stroke admissions to the Norfolk and Norwich University Hospitals, a large tertiary centre in England covering an area with a catchment population of 790,000 in 2003 and 876,000 in 2016 [9]. Ethical approval was obtained from the Newcastle and Tyneside National Health Service Research Ethics Committee (17/NE/0277) as a research database, which does not require individual patient consent. The protocol of our study was approved by the steering committee of the NNSTR. Selection criteria as well as data collection methods have been described previously [8]. All patients in the current study were admitted between January 2003 and December 2016, and followed up until June 2017. The data for this study are available from the corresponding author upon reasonable request.

### Predictors

Candidate predictors were chosen based on the literature. This was followed by a manual backward elimination process which excluded candidate predictors which did not exhibit a statistically significant relationship with the log hazard of mortality, as described in detail below in the ‘Cox proportional-hazards model’ section. Candidate predictors included in the elimination process were age [10], sex [10], pre-stroke disability (measured using the modified Rankin Score), type of stroke [11], haemoglobin [12], sodium [13], white blood cell count [14], as well as major comorbidities such as ischaemic heart disease [10], atrial fibrillation [9], cancers [15], hypertension [16], chronic obstructive pulmonary disease [17], liver disease [18], peripheral vascular disease [19], heart failure [20, 21] and diabetes [10] eGFR [22]. The candidate predictors were measured on admission unless specified otherwise. The indicator of renal function in our model, eGFR, was calculated using the imputed creatinine values and the Chronic Kidney Disease Epidemiology Collaboration formula [23]. Pre-existing co-morbidity status on admission was identified from International Classification of Diseases-Tenth Revision codes based on clinical findings and retrieved from the hospital administration database (Supplementary Table 1 in the online-only Data Supplement). Continuous predictors that were found not to have a normal distribution (white blood cell count and sodium) were winsorized to the 99th percentile to reduce the influence of extreme outliers on the results.

### Outcome

The outcome of interest was all-cause mortality within the 10 years after ischaemic stroke. Discharge status (alive or deceased) was recorded in the hospital. Mortality information after discharge was obtained by record linkage with the Office of National Statistics database which captures all deaths in the UK.

### Handling of missing data

We performed a logistic regression (Supplementary Table 8) to explore the differences between patients with missing data and those without any missing data.

All predictors with missingness under 15% were considered in the initial model.

It has been recommended that the number of imputed datasets should be equal or higher than the percentage of cases with at least one missing variable. Based on the proportion of cases with at least one variable with missing data [24] we imputed 20 datasets. Based on the characteristics of the patients with missing data and those without, the data were deemed to be likely to be missing at random [24]. A multiple imputation by chained equations algorithm with 20 imputed datasets was thus implemented in R version 3.6.3 (R package *mice* [25]) using a predictive mean matching algorithm drawing from five nearest neighbours. Coronary heart disease, diabetes, congestive heart failure, chronic kidney disease, atrial fibrillation, cancers, dementia, COPD, liver disease, hyperlipidaemia, PVD, age, sex and the Nelson–Aalen cumulative hazard were included as predictors.

### Statistical analysis

Data were analysed using R version 3.6.3 (R Foundation for Statistical Computing, Vienna, Austria) and Stata 15.1 SE (StataCorp 2017, Stata Statistical Software: Release 15, College Station, TX: StataCorp LLC). The R *rms* package [26] was employed to internally validate the score and create the nomogram, whilst the *hmisc* package [27] was employed to perform the Cox proportional-hazards model. The median follow-up time was calculated using the reverse Kaplan–Meier method [28].

### Cox proportional-hazards model

A Cox proportional hazards model was used to obtain regression coefficients. All the candidate predictors were added and then through a manual backward elimination process the non-significant predictors were removed. Only significant predictors were included in the final model (*p* < 0.05). Given that blood measurement data (haemoglobin, white blood count, and sodium) may not exhibit a linear relationship with the outcome, these predictors were added to the model as restricted cubic splines (RCS) if this parametrisation was found to improve model fit. The transformation that resulted in the model with the lowest Akaike Information Criterion (AIC) was chosen for each predictor. Stroke type (OCSP classification), pre-stroke modified Rankin score and estimated glomerular filtration rate (eGFR) were included as categorical variables. The eGFR was calculated using the imputed creatinine values and categorised as: > 90, 60–90, 45–60, 30–45, 15–30, <  = 15 (> 90 as the reference category), given the previously described predictive inaccuracy of eGFR at values > 90 [29]. For stroke type, the designated reference category was LACS.

### Nomogram development

The prediction model was developed and validated was then converted into a nomogram to allow clinicians to easily calculate the predicted probability of 10-year mortality in stroke patients. The nomogram was created based on the coefficients of the Cox proportional-hazards model. Each predictor value is represented on a separate axis and the amount of corresponding points is noted from the ‘Points’ axis. The total of the points from all predictors (Total Point axis) then maps to a predicted 10-year mortality at the bottom of the nomogram (10-year Mortality axis).

### Internal validation

Nagelkerke’s *R*^2^ was used to measure the proportion of variation of the outcome that can be explained by the regression model and predictors [30]. To assess the ability of the model to discriminate between patients at high and low risk of mortality, we used Harrell’s C-statistic. We internally validated the prediction model using 500 bootstrapped samples from the dataset to obtain optimism-adjusted c-statistic and calibration slope. This method involves generating samples of the same size as the original dataset with replacement [26]. New final models were developed in each of the bootstrap samples and their performance (C-statistic and calibration curve) was assessed (bootstrap performance). Each of the models was then applied to the original dataset and the same performance was assessed (test performance). The average difference in the bootstrap and test performance is the ‘optimism’ in performance of the original model. Optimism-adjusted performance is estimated as performance in the original dataset minus the ‘optimism’. This is an estimate of internal validity, reflecting validation for the underlying population where the data originated from. Hazard regression was used to estimate the relationship between the predicted survival probability at 10 years and the observed outcomes to derive a calibration curve [31, 32]. The bootstrap process de-biased the estimates to correct for overfitting. The calibration slope represents the gradient of a linear calibration curve. Its value indicates whether the model is overfitting (slope < 1) or underfitting (slope > 1). For easier visualisation of the score performance, we also plotted Kaplan–Meier curves by score quintiles and a histogram of the total points’ distribution across the study sample.

### Data availability

The data underlying this study are available from the corresponding author upon reasonable request.

## Results

### Patient population

A total of 10,841 first-ever ischaemic stroke admissions between Jan 2003 and Dec 2016 were initially extracted from the NNSTR. Patients with missing follow-up data (*n* = 81), those with missing discharge data (*n* = 221) and those aged under 45 (*n* = 175) were excluded due to the likely different underlying mechanisms of disease in younger patients. Therefore, a total of 10,366 ischaemic stroke patients aged 45 years and over were included in the analysis (Supplementary Fig. 1).

Table 1 presents summary characteristics on admission for the entire cohort. Mean age was 78.5 years (standard deviation 10.9 years). There were 5409 females (52%). A total of 3534 (38.0%) patients suffered a Partial Anterior Circulation Stroke (PACS), 2401 (25.8%) of them had a Lacunar Stroke (LACS), 1830 (19.7%) patients had a Total Anterior Circulation Stroke (TACS) and 1533 (16.5%) patients had a Posterior Circulation Stroke (POCS). A total of 2916 patients (28.1%) suffered from coronary heart disease and 6377 (61.5%) from hypertension. Heart failure was diagnosed in 1481 (14.3%) patients, atrial fibrillation in 3393 (32.7%) patients, cancers in 1660 (16.0%) patients, liver disease in 156 (1.5%) patients and peripheral vascular disease in 437 (4.2%) patients. Over the 10 years of follow-up, 4879 patients died (47.1%). Baseline 10-year survival was 44%. Median (95% CI) follow-up was 5.47 (5.35–5.58) years.

**Table 1:** Patient characteristics

Characteristic	All (*n* = 10,366)
Age, mean (SD)	78.4 (10.9)
10-year mortality, *N* (%)	4879 (47.1)
Haemoglobin, mean (SD)	134.4 (19.6)
WBC, median (IQR)	8.8 (7.1–11.3)
Creatinine, mean (SD)	1.1(0.5)
eGFR, *N* (%)	
< = 15	128 (1.3)
15–30	595 (5.9)
30–45	1319 (13.1)
45–60	2089 (20.7)
60–90	4875 (48.2)
> 90	1102 (10.9)
Sodium, median (IQR)	139 (136–141)
Sex	
Male, *N* (%)	4957 (47.8)
Female, *N* (%)	5409 (52.2)
OCSP classification	
PACS, *N* (%)	3534 (38.0)
LACS, *N* (%)	2401 (25.8)
TACS, *N* (%)	1830 (19.7)
POCS, *N* (%)	1533 (16.5)
Modified Rankin Scale (on admission)	
0, *N* (%)	6085 (62.4)
1, *N* (%)	1250 (12.8)
2, *N* (%)	835 (8.6)
3, *N* (%)	942 (9.7)
4, *N* (%)	453 (4.6)
5, *N* (%)	193 (2.0)
Coronary heart disease, *N* (%)	2916 (28.1)
Heart failure, *N* (%)	1481 (14.3)
Atrial fibrillation, *N* (%)	3393 (32.7)
Cancers, *N* (%)	1660 (16.0)
Hypertension, *N* (%)	6377 (61.5)
Liver disease, *N* (%)	156 (1.5)
Peripheral vascular disease, *N* (%)	437 (4.2)

*SD* standard deviation, *IQR* inter-quartile range, *OCSP* oxfordshire community stroke project, *eGFR* estimated glomerular filtration rate, *PACS* partial anterior circulation stroke, *LACS* lacunar circulation stroke, *TACS* total anterior circulation stroke, *POCS* posterior circulation stroke

### Missing data

The following six candidate predictors had missing data (ranging from 2.2 to 10.3%): OCSP stroke classification (10.3%), pre-stroke modified Rankin score (5.9%), haemoglobin (4.9%), white blood cell count (2.2%), sodium (2.6%), and creatinine (2.5%). Supplementary Tables 2–7 detail the characteristics of the included cohort, stratified by whether each variable in question had missing data. Patients with liver disease, patients without hypertension, as well as older patients were more likely to have missing data.

CRP, cholesterol and serum glucose were not included in the model since they were missing for a high proportion of patients (17.16%, 36.1%, and 24.19%, respectively).

### Cox proportional-hazards model

Candidate predictors included in the final Cox proportional hazards model were age [10], sex [10], pre-stroke disability (measured using the modified Rankin Score), type of stroke [11], haemoglobin [12], sodium [13], white blood cell count [14], ischaemic heart disease [10], atrial fibrillation [9], cancers [15], hypertension [16], chronic obstructive pulmonary disease [17], liver disease [18], peripheral vascular disease [19], heart failure [20, 21] and eGFR [22]. Diabetes was the only candidate predictor that was excluded from the final model.

Table 2 details the association between each predictor and mortality within 10 years as hazard ratios and parameter estimates from the Cox proportional-hazards model. The baseline survival at 10 years was 0.44. Patients with TACS were almost 3 times more likely to die compared those with LACS, hazard ratio (95% confidence interval) (HR, 95% CI): (2.87, 2.62–3.14). Those with eGFR below 15 were almost twice more likely to die after stroke compared to those with eGFR over 90 (1.97, 1.55–2.52). Out of all the comorbidities included, liver disease had the strongest effect on stroke mortality, with a hazard ratio of (1.50, 1.20–1.87). Hypertension was inversely associated with 10-year mortality HR: (0.77, 0.72–0.82). A 1-year increase in age was associated with a 4% increased hazard of mortality HR: (1.04, 1.04–1.05). Hazard ratios for haemoglobin, sodium and white blood cell count values were computed using RCSs and are shown in Supplementary Fig. 2.

**Table 2:** Results of the multivariable Cox proportional-hazards model of 10-year stroke mortality

Variable	HR (95% CI)	Log HR (95% CI)
Age	1.04 (1.04, 1.05)	0.042 (0.039, 0.046)
Sex (F:M)	0.87 (0.82,0.93)	− 0.140 (− 0.202, − 0.077)
OCSP classification		
LACS	Reference	Reference
PACS	1.20 (1.10, 1.30)	0.182 (0.099, 0.265)
TACS	2.87 (2.62, 3.14)	1.055 (0.965, 1.145)
POCS	1.29 (1.16, 1.44)	0.257 (0.152, 0.362)
eGFR		
< = 15	1.97 (1.55, 2.52)	0.680 (0.438, 0.923)
15–30	1.61 (1.36, 1.91)	0.475 (0.304, 0.646)
30–45	1.34 (1.15, 1.57)	0.294 (0.138, 0.451)
45–60	1.12 (0.96, 1.30)	0.110 (− 0.041, 0.261)
60–90	1.04 (0.91, 1.20)	0.043 (− 0.098, 0.184)
> 90	Reference	Reference
Modified Rankin Scale (on admission)	1.20(1.17, 1.22)	0.181 (0.160, 0.202)
Atrial fibrillation	1.17 (1.10, 1.24)	0.153 (0.092, 0.214)
Coronary heart disease	1.09 (1.02, 1.16)	0.083 (0.018, 0.148)
Congestive heart failure	1.24 (1.15, 1.34)	0.216 (0.138, 0.295)
Cancers	1.37 (1.27, 1.47)	0.312 (0.239, 0.385)
Hypertension	0.77 (0.72, 0.82)	− 0.262 (− 0.322, − 0.202)
Chronic obstructive pulmonary disease	1.13 (1.03, 1.25)	0.125 (0.025, 0.225)
Liver disease	1.50 (1.20, 1.87)	0.405 (0.184, 0.625)
Peripheral vascular disease	1.39 (1.23, 1.57)	0.329 (0.204, 0.454)
Haemoglobin	^a^	
RCS1		− 0.152 (− 0.183, 0.886)
RCS2		− 0.056 (− 0.085, 0.973)
RCS3		− 0.019 (− 0.046, 1.008)
RCS4		− 0.029 (− 0.057, 0.998)
RCS5		− 0.020 (− 0.049, 1.010)
RCS6		− 0.006 (− 0.037, 1.025)
WBC	^a^	
RCS1		0.013 (− 0.015, 1.042)
RCS2		− 0.045 (− 0.072, 0.982)
RCS3		− 0.042 (− 0.069, 0.986)
RCS4		0.033 (0.005, 1.062)
RCS5		− 0.009 (− 0.038, 1.020)
Sodium	^a^	
RCS1		0.206 (0.178, 1.265)
RCS2		0.002 (− 0.025, 1.030)
RCS3		0.069 (0.042, 1.100)
RCS4		− 0.021 (− 0.050, 1.008)
RCS5		− 0.009 (− 0.039, 1.020)
RCS6		− 0.035 (− 0.063, 0.993)

*HR* hazard ratio, *OCSP* oxfordshire community stroke project, *eGFR* estimated glomerular filtration rate, *PACS* partial anterior circulation stroke, *LACS* Lacunar Circulation Stroke, *TACS* total anterior circulation stroke, *POCS* posterior circulation stroke, *RCS* restricted cubic spline

^a^Variables were presented as restricted cubic splines (see Supplementary Fig. 2). See “Results” for hazard ratios at different values of these predictors. Baseline survival was 44%

### Internal validation

**Fig. 1:**
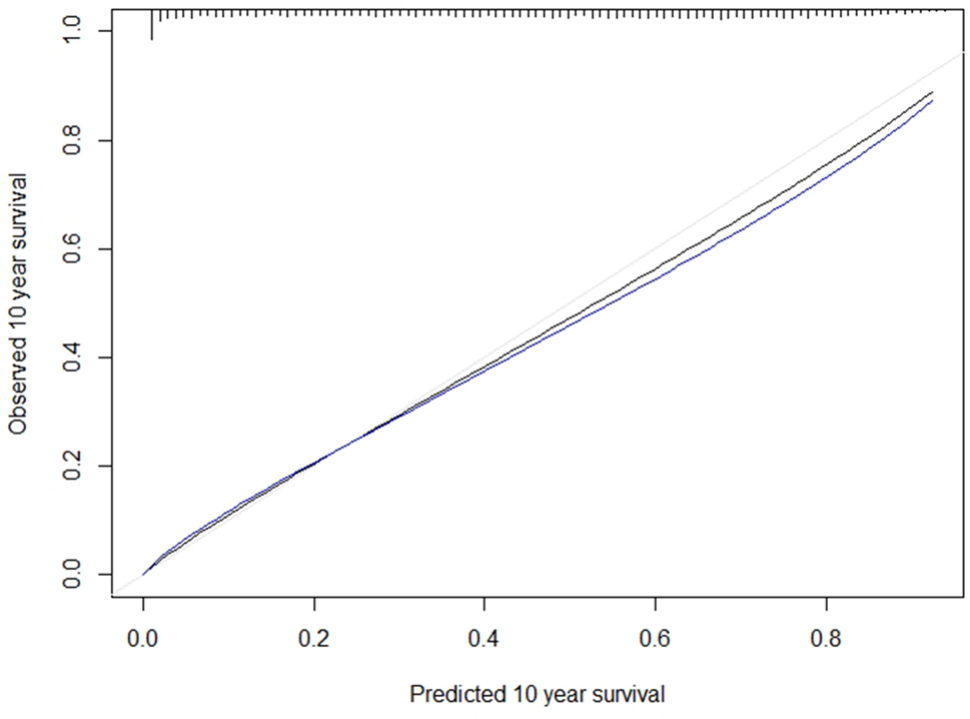
Calibration plot—the observed 10-year survival is plotted against the predicted 10-year survival in groups of 500 patients. The black line is the calibration in the original dataset estimated by hazard regression. The blue line is the optimism-corrected hazard regression smoothed curves. The gray line is a reference line representing perfect calibration

The *R*-squared for the model was 0.32 whilst the C-statistic was 0.76 which is recognised as ‘fair’ discrimination ability [33]. After internal validation, the optimism-adjusted R-squared slightly reduced to 0.31 whilst the C-statistic remained the same. The calibration slope was 0.98 indicating good model fit. The relationship between the predicted and observed probabilities was also assessed visually using a calibration plot and showed good agreement (Fig. 1). The blue line on the plot represents the bootstrap bias-corrected calibration curve and displays evidence of slight overprediction for good prognosis patients. However, for poor prognosis patients (survival < 40%), the model appears to be accurate.

**Fig. 2:**
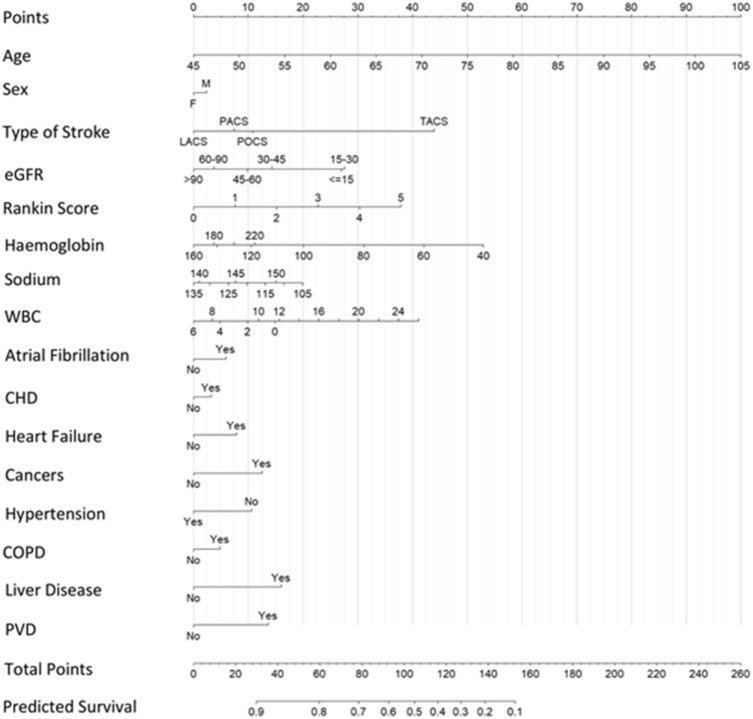
Nomogram. A nomogram is a tool used for calculating probabilities in predictive models using a visual representation. The nomogram was created based on the Cox proportional-hazards model coefficients (Table 2 and Supplementary Fig. 2). Each variable value is represented on a separate axis. Each variable’s value should be found on the variable axis, then the amount of corresponding points should be noted from the ‘Points’ axis. The total of all these scores (Total Point axis) can then be used to determine the predicted 10-year mortality (10-year Mortality axis)

Figure 2 details the resulting score nomogram based on the results of the Cox proportional-hazards model. For example, a female 60-year-old patient with haemoglobin level of 120, sodium of 135, white blood count of 6.5, eGFR of 45, with pre-stroke modified Rankin score of 1 and history of AF and no other comorbidities, who suffered a partial anterior circulation stroke, would receive 71.66 points. This corresponds to 10-year survival of 0.74.

Supplementary Fig. 4 details the distribution of the total score points across the study population, calculated using the provided nomogram. The total score points were normally distributed across the study population with mean (SD): 113 (38.6), Min = 11.54 and Max = 250.36.

Supplementary Fig. 5 details the observed 10-year survival curves, stratified by score quintiles (Fifth 1: 11.5–79.5, Fifth 2: 79.4, 100.9, Fifth 3: 100.9–120.9, Fifth 4:120.9–145.8, Fifth 5: 145.8–250.4). The score discriminates well between strata according to their risk of death within 10 years.

## Discussion

To the best of our knowledge, this is the first study to develop and internally validate a nomogram to predict 10-year mortality using point of care data for patients with an ischaemic stroke. Using a large UK regional registry of consecutive stroke admissions with long-term post-discharge follow-up data, we developed a prediction model that uses co-morbidities, laboratory results as well as age and sex of the patients and stroke type. Our score, presented as a nomogram for clinical use, is a simple tool that can be used to predict long-term patient mortality and includes only routinely collected variables during initial routine work-up on acute stroke admissions, rendering them available in most healthcare facilities. It has numerous applications in practice, including informing discussions and planning with the patient and families and providing the prognosis for patients. Additionally, future studies can use the score as a benchmark for interventions.

Numerous stroke mortality scores have been previously developed. Outcomes considered in such scores include mortality and recurrence, as well as functional outcomes measured by mRS (modified Rankin Score) and length-of-stay in the hospital. They have been analysed and compared in several reviews [4–6]. However, so far, none of them predicts mortality as far as 10 years post-stroke. Furthermore, none of them is commonly used in clinical practice. One of the possible reasons is the complexity of calculating the scores either due to complicated formulae or incorporating the neuroimaging results. Furthermore, some of the scores require specialist knowledge, which can make them less likely to be used by general clinicians.

Whilst research into 10-year ischaemic stroke mortality predictors is limited due to most studies lacking such long follow-up, it has been previously shown that predictors of short- and long-term stroke mortality predictors can differ [34]. The difference is particularly visible when comorbidities and variables associated with atherosclerotic vascular disease are considered (such as peripheral artery disease), as they seem to predict long-term, but not short-term mortality [34]. Most prediction models for short-term ischaemic stroke mortality focus on acute baseline details such as glucose levels on admission, time from onset to presentation, GCS (Glasgow Coma Scale), level of consciousness and stroke subtype [35]. While some of these factors may predict long-term outcomes, longer term prediction models rely more heavily on comorbid conditions [34]. In our score, candidate predictors such as ischaemic heart disease, atrial fibrillation, cancers, hypertension, COPD, liver disease, peripheral vascular disease and heart failure were included in the final model.

Stroke mortality was a common outcome predicted by previous scores, which included inpatient [35], 7 days [35], 1 month [36], 3 months [37] 6 months [38], 1 year [36, 39, 40], 2 years [41] and 5 years [42] mortality. The longest term mortality study [43] predicted death at 10 years or more, but this study only included patients with Transient Ischaemic Attacks or minor strokes. One of the scores with similar outcome and patient population (ischaemic stroke) is the ASTRAL score [42]. It was initially created to predict 3 months mortality and was also found to predict mortality up to 5 years. The predictors included were age, acute glucose, visual field defect, level of consciousness, time from the onset of symptoms and stroke severity (NIHSS). However, the ASTRAL score requires availability of measurement of the time from stroke onset, which can sometimes be difficult to obtain and thus not available in all patients.

A review of acute stroke prediction models [6] such as iSCORE, SOAR, PLAN and THRIVE amongst others, concluded that practical aspects of the score can be potentially a limiting factor in the clinical usage of the scores, as the majority of them have a good predictive accuracy and have been externally validated. Data unavailability and limited resources can be a potential obstacle in wider adoption of these scores, which can be the case with some of the models including biomarkers [44] or those using imaging data [45].

Our score and nomogram have numerous strengths. This 10-year ischaemic stroke score provides quick way for clinicians to estimate predicted survival of their patients at the time of stroke presentation. Such information can be useful in several circumstances, including in making appropriate decisions regarding secondary prevention and facilitating discussions with patients and their relatives/carers. Furthermore, understanding long-term survival in this patient population is relevant and pertinent to health care planning and policy. It has been developed on a large sample (10,366 patients) with robust ascertainment of co-morbidities and follow-up data through data linkage with the UK National Health Service system [9]. Furthermore, it only contains data available on admission and does not require any specialist investigations. Visualisation of the score in a form of a nomogram increases the potential of uptake in clinical practice especially in conjunction with future CV risk prediction scores and can help clinicians to risk stratify for appropriate intervention strategies. It does not require stroke specialist knowledge, which makes it more generally useful for other healthcare professionals.

We also acknowledge certain limitations. The inverse association between hypertension and 10-year mortality in our model was unexpected. This is likely to be driven by residual confounders, such as anti-hypertensive treatment, data on which were unavailable in our registry. Another factor affecting this association may be stroke severity. The NIHSS scale recorded in our database had a high proportion of missing values and could not be incorporated in the model. However, we utilised the OCSP stroke classification as a proxy for stroke severity [46]. Further research should assess whether the addition of treatment data and NIHSS to our score would increase the predictive ability of the score using. We were unable to adjust for treatment effect but it is complex and unrealistic to fully account for this given treatment variation over time. The same principle applies with regard to stroke severity (NIHSS), some relevant blood results (glucose, cholesterol and C-reactive protein) and severity of comorbidities. External validation on a different dataset is also needed to assess its generalisability in a global setting.

## Conclusion

In conclusion, we have developed the first 10-year stroke mortality score, taking into account a wide range of comorbid factors and blood parameters available on admission. We also provide a nomogram to facilitate application at the point of care. Further external validation on an independent dataset is required to ensure generalisation to different ethnicities and healthcare settings.

## Supplementary Information

Below is the link to the electronic supplementary material.

Supplementary file1 (DOCX 672 KB)

## References

[1] Katan M, Luft A (2018). Global burden of stroke. Semin Neurol.

[2] Johnson W, Onuma O, Owolabi M, Sachdev S (2016). Stroke: a global response is needed. Bull World Health Organ.

[3] IHME (2018) I. for H. M. and E. United Kingdom Profile. vol. 2019 http://www.healthdata.org/united-kingdom

[4] Drozdowska BA, Singh S, Quinn TJ (2019). Thinking about the future: a review of prognostic scales used in acute stroke. Front Neurol.

[5] Counsell C, Dennis M (2001). Systematic review of prognostic models in patients with acute stroke. Cerebrovasc Dis.

[6] Fahey M, Crayton E, Wolfe C, Douiri A (2018). Clinical prediction models for mortality and functional outcome following ischemic stroke: a systematic review and meta-analysis. PLoS ONE.

[7] Hannerz H, Nielsen ML (2001). Life expectancies among survivors of acute cerebrovascular disease. Stroke.

[8] Bettencourt-Silva J, De La Iglesia B, Donell S, Rayward-Smith V (2012). On creating a patient-centric database from multiple Hospital Information Systems. Methods Inf Med.

[9] Pana TA (2019). Individual and combined impact of heart failure and atrial fibrillation on ischemic stroke outcomes. Stroke.

[10] Ronning OM, Stavem K (2012). Predictors of mortality following acute stroke: a cohort study with 12 years of follow-up. J Stroke Cerebrovasc Dis.

[11] Bamford J, Sandercock P, Dennis M, Burn J, Warlow C (1991). Classification and natural history of clinically identifiable subtypes of cerebral infarction. Lancet.

[12] Barlas RS (2016). Impact of hemoglobin levels and anemia on mortality in acute stroke: analysis of UK regional registry data, systematic review, and meta-analysis. J Am Heart Assoc.

[13] Soiza RL (2015). Hyponatremia predicts mortality after stroke. Int J Stroke.

[14] Zheng X (2018). Prognostic value of white blood cell in acute ischemic stroke patients. Curr Neurovasc Res.

[15] Grazioli S (2018). Cancer-associated ischemic stroke: a retrospective multicentre cohort study. Thromb Res.

[16] Maier B (2017). Mortality and disability according to baseline blood pressure in acute ischemic stroke patients treated by thrombectomy: a collaborative pooled analysis. J Am Heart Assoc.

[17] Lekoubou A, Ovbiagele B (2017). Prevalance and influence of chronic obstructive pulmonary disease on stroke outcomes in hospitalized stroke patients. eNeurologicalSci.

[18] Parikh NS, Merkler AE, Schneider Y, Navi BB, Kamel H (2017). Discharge disposition after stroke in patients with liver disease. Stroke.

[19] Meves SH (2010). Peripheral arterial disease as an independent predictor for excess stroke morbidity and mortality in primary-care patients: 5-year results of the getABI study. Cerebrovasc Dis.

[20] Collins TC (2003). Short-term, intermediate-term, and long-term mortality in patients hospitalized for stroke. J Clin Epidemiol.

[21] Pana TA (2019). Impact of heart failure on stroke mortality and recurrence. Heart Asia.

[22] Vart P (2019). Estimated glomerular filtration rate and risk of poor outcomes after stroke. Eur J Neurol.

[23] Levey AS (2009). A new equation to estimate glomerular filtration rate. Ann Intern Med.

[24] White IR, Royston P, Wood AM (2011). Multiple imputation using chained equations: Issues and guidance for practice. Stat Med.

[25] Van Buuren S (2018) Flexible imputation of missing data, 2nd edn. Chapman and Hall/CRC

[26] Harrel F (2015) Regression modeling strategies. Springer

[27] Alzola C, Harrel F (2006) An introduction to S and the Hmisc and design libraries

[28] Shuster JJ (1991). Median follow-up in clinical trials. J Clin Oncol.

[29] Stevens LA, Coresh J, Greene T, Levey AS (2006). Assessing kidney function–measured and estimated glomerular filtration rate. N Engl J Med.

[30] Nagelkerke NJD (1991). A note on a general definition of the coefficient of determination. Biometrika.

[31] Kooperberg C, Stone CJ, Truong YK (1995). Hazard Regression. J Am Stat Assoc.

[32] Harrel F (2006). Regression modeling strategies: with applications to linear models, logistic regression, and survival analysis (Springer Series in Statistics).

[33] Carter JV, Pan J, Rai SN, Galandiuk S (2016). ROC-ing along: evaluation and interpretation of receiver operating characteristic curves. Surgery.

[34] Koton S, Tanne D, Green MS, Bornstein NM (2010). Mortality and predictors of death 1 month and 3 years after first-ever ischemic stroke: data from the first National Acute Stroke Israeli Survey (NASIS 2004). Neuroepidemiology.

[35] Kwok CS (2013). The SOAR stroke score predicts inpatient and 7-day mortality in acute stroke. Stroke.

[36] Saposnik G (2011). IScore: a risk score to predict death early after hospitalization for an acute ischemic stroke. Circulation.

[37] Song B (2019). A COACHS nomogram to predict the probability of three-month unfavorable outcome after acute ischemic stroke in Chinese patients. Cerebrovasc Dis.

[38] Sun C (2019). A NADE nomogram to predict the probability of 6-month unfavorable outcome in Chinese patients with ischemic stroke. BMC Neurol.

[39] Liljehult J, Christensen T, Christensen KB (2020). Early prediction of one-year mortality in ischemic and haemorrhagic stroke. J Stroke Cerebrovasc Dis.

[40] Ling X, Shen B, Li K, Si L, Yang X (2019). Development of a prediction model for 1-year poor prognosis in patients with acute ischemic stroke. J Investig Med.

[41] Vitturi BK, Gagliardi RJ (2020). Use of CHADS2 and CHA2DS2-VASc scores to predict prognosis after stroke. J Rev Neurol.

[42] Papavasileiou V (2013). ASTRAL score predicts 5-year dependence and mortality in acute ischemic stroke. Stroke.

[43] van Wijk I (2005). Long-term survival and vascular event risk after transient ischaemic attack or minor ischaemic stroke: a cohort study. Lancet.

[44] De Marchis GM (2019). A novel biomarker-based prognostic score in acute ischemic stroke: The CoRisk score. Neurology.

[45] Tateishi Y (2019). A score using left ventricular diastolic dysfunction to predict 90-day mortality in acute ischemic stroke: the DONE score. J Neurol Sci.

[46] Sprigg N (2007). Stroke severity, early recovery and outcome are each related with clinical classification of stroke: data from the ‘Tinzaparin in Acute Ischaemic Stroke Trial’ (TAIST). J Neurol Sci.

