# Supplementary material for: Predicting 10-year stroke mortality: development and validation of a nomogram
Source: Acta Neurol Belg. 2021 Aug 18;122(3):685–93. doi: 10.1007/s13760-021-01752-9 (PMC9170668; doi:10.1007/s13760-021-01752-9)
Supplement: Supplementary file 1 — Supplementary file1 (DOCX 672 KB) [file 13760_2021_1752_MOESM1_ESM.docx]

**Supplementary Material**

**Supplementary Tables**

**Supplementary Table 1**. Confounding co-morbidities and their corresponding International Statistical Classification of Diseases and Related Health Problems 10th Revision (ICD-10) codes

| **Co-morbidity** | **ICD-10 code** |
| --- | --- |
| Atrial Fibrillation | I48 |
| Coronary Heart Disease | I20 - I25 |
| Heart Failure | I50 |
| Cancers | C00 - C97 |
| Hypertension | I10 - I15 |
| Chronic Obstructive Pulmonary Disease | J40 - J44, J47 |
| Liver Disease | K70 - K77 |
| Peripheral Vascular Disease | I73.9 |

**Supplementary Table 2**. Patient characteristics stratified by whether the data for **sodium levels** were missing

| Variable | Total | Missing | Non-missing | *P*-value |
| --- | --- | --- | --- | --- |
| Age, mean (SD) | 78.48 (10.90) | 78.51 (10.88) | 77.44 (11.52) | 0.111 |
| Female, N(%) | 5409 (52.2) | 125 (46.6%) | 5284 (52.3%) | 0.066 |
| Pre-stroke mRS, median (IQR) | 0.0 (0.0-1.0) | 0.0 (0.0-1.0) | 0.0 (0.0-1.0) | 0.261 |
| Death, N(%) | **4887 (47.1)** | **97 (36.2)** | **4790 (47.4)** | **<0.001** |
| OCSP classification |  |  |  | **0.005** |
| PACS, N(%) | 3534 (34.1) | 89 (37.3) | 3445 (34.1) |  |
| LACS, N(%) | 2401 (23.2) | 53 (37.3) | 2348 (23.3) |  |
| TACS, N(%) | 1830 (17.7) | 34 (37.3) | 1796 (17.8) |  |
| POCS, N(%) | 1533 (14.8) | 52 (37.3) | 1481 (14.7) |  |
| Coronary Heart Disease, N(%) | **2916 (28.1)** | **48 (17.9)** | **2868 (28.4)** | **<0.001** |
| Peripheral Vascular Disease, N(%) | 437 (4.2) | 11 (4.1) | 426 (4.2) | 0.927 |
| Hypertension, N(%) | **6377 (61.5)** | **140 (52.2)** | **6237 (61.8)** | **0.002** |
| Diabetes, N(%) | 1876 (18.1) | 51 (19.0) | 1825 (18.1) | 0.688 |
| Chronic Obstructive Pulmonary Disease, N(%) | **865 (8.3)** | **13 (4.9)** | **852 (8.4)** | **0.036** |
| Cancers, N(%) | 1660 (16.0) | 35 (13.1) | 1625 (16.1) | 0.182 |
| Liver Disease, N(%) | 156 (1.5) | 3 (1.1) | 153 (1.5) | 0.599 |
| Plasma glucose on admission, median (IQR) | 6.3 (5.5-7.9) | 6.3 (5.5-7.9) | 6.3 (5.3-10.9) | 0.677 |
| Creatinine on admission, median (IQR) | 0.9 (0.8-1.2) | 0.9 (0.8-1.2) | 0.8 (0.6-1.0) | 0.073 |
| C-reactive protein on admission, median (IQR) | **12.0 (5.0-37.0)** | **12.0 (5.0-37.0)** | **4.0 (3.0-9.0)** | **<0.001** |
| WBC on admission, median (IQR) | 8.8 (7.1-11.3) | 8.8 (7.1-11.3) | 8.3 (6.8-11.0) | 0.123 |
| Sodium on admission, mean (SD) | - | - | - | - |
| Cholesterol on admission, mean (SD) | 4.85 (1.31) | 4.85 (1.31) | 5.19 (1.20) | 0.079 |
| Haemoglobin on admission, mean (SD) | **134.43 (19.58)** | **134.40 (19.60)** | **140.79 (15.85)** | **0.035** |

SD – standard deviation, IQR – inter-quartile range ; mRS – modified Rankin Scale ; OCSP – Oxfordshire Community Stroke Project; PACS – partial anterior circulation stroke; LACS – lacunar stroke; TACS – total anterior circulation stroke; POCS – posterior circulation stroke; WBC – white blood cell count

Statistically significant results are highlighted in **bold**.

**Supplementary Table 3**. Patient characteristics stratified by whether the data for **white blood count levels** were missing

| Variable | Total | Missing | Non-missing | *P*-value |
| --- | --- | --- | --- | --- |
| Age, mean (SD) | 78.48 (10.90) | 78.48 (10.89) | 78.73 (11.05) | 0.729 |
| Female, N(%) | 5409 (52.2) | 112 (49.1%) | 5297 (52.2%) | 0.350 |
| Pre-stroke mRS, median (IQR) | 0.0 (0.0-1.0) | 0.0 (0.0-1.0) | 0.0 (0.0-1.0) | 0.795 |
| Death, N(%) | **4887 (47.1)** | **91 (39.9)** | **4796 (47.3)** | **0.027** |
| OCSP classification |  |  |  | **0.005** |
| PACS, N(%) | 3534 (34.1) | 71 (37.2) | 3463 (34.2) |  |
| LACS, N(%) | 2401 (23.2) | 42 (37.2) | 2359 (23.3) |  |
| TACS, N(%) | 1830 (17.7) | 36 (37.2) | 1794 (17.7) |  |
| POCS, N(%) | 1533 (14.8) | 40 (37.2) | 1493 (14.7) |  |
| Coronary Heart Disease, N(%) | **2916 (28.1)** | **42 (18.4)** | **2874 (28.3)** | **<0.001** |
| Peripheral Vascular Disease, N(%) | 437 (4.2) | 7 (3.1) | 430 (4.2) | 0.384 |
| Hypertension, N(%) | **6377 (61.5)** | **121 (53.1)** | **6256 (61.7)** | **0.008** |
| Diabetes, N(%) | 1876 (18.1) | 46 (20.2) | 1830 (18.1) | 0.410 |
| Chronic Obstructive Pulmonary Disease, N(%) | 865 (8.3) | 12 (5.3) | 853 (8.4) | 0.089 |
| Cancers, N(%) | 1660 (16.0) | 37 (16.2) | 1623 (16.0) | 0.929 |
| Liver Disease, N(%) | 156 (1.5) | 3 (1.3) | 153 (1.5) | 0.813 |
| Plasma glucose on admission, median (IQR) | 6.3 (5.5-7.9) | 6.3 (5.5-7.9) | 6.0 (5.1-7.4) | 0.473 |
| Creatinine on admission, median (IQR) | 0.9 (0.8-1.2) | 0.9 (0.8-1.2) | 0.9 (0.8-1.1) | 0.366 |
| C-reactive protein on admission, median (IQR) | **12.0 (5.0-37.0)** | **12.0 (5.0-37.0)** | **5.0 (2.0-6.0)** | **0.004** |
| WBC on admission, median (IQR) | - | - | - | - |
| Sodium on admission, mean (SD) | 138.24 (4.08) | 138.24 (4.08) | 138.00 (2.69) | 0.772 |
| Cholesterol on admission, mean (SD) | 4.85 (1.31) | 4.85 (1.31) | 4.91 (1.19) | 0.858 |
| Haemoglobin on admission, mean (SD) | 134.43 (19.58) | 134.43 (19.58) | - |  |

SD – standard deviation, IQR – inter-quartile range ; mRS – modified Rankin Scale ; OCSP – Oxfordshire Community Stroke Project; PACS – partial anterior circulation stroke; LACS – lacunar stroke; TACS – total anterior circulation stroke; POCS – posterior circulation stroke; WBC – white blood cell count

Statistically significant results are highlighted in **bold**.

**Supplementary Table 4**. Patient characteristics stratified by whether the data for **haemoglobin levels** were missing

| Variable | Total | Missing | Non-missing | *P*-value |
| --- | --- | --- | --- | --- |
| Age, mean (SD) | 78.48 (10.90) | 78.52 (10.84) | 77.74 (11.85) | 0.113 |
| Female, N(%) | 5409 (52.2) | 257 (50.6%) | 5152 (52.3%) | 0.462 |
| Pre-stroke mRS, median (IQR) | 0.0 (0.0-1.0) | 0.0 (0.0-1.0) | 0.0 (0.0-1.0) | 0.305 |
| Death, N(%) | **4887 (47.1)** | **154 (30.3)** | **4733 (48.0)** | **<0.001** |
| OCSP classification |  |  |  | 0.495 |
| PACS, N(%) | 3534 (34.1) | 179 (41.3) | 3355 (34.0) |  |
| LACS, N(%) | 2401 (23.2) | 126 (41.3) | 2275 (23.1) |  |
| TACS, N(%) | 1830 (17.7) | 82 (41.3) | 1748 (17.7) |  |
| POCS, N(%) | 1533 (14.8) | 78 (41.3) | 1455 (14.8) |  |
| Coronary Heart Disease, N(%) | 2916 (28.1) | 129 (25.4) | 2787 (28.3) | 0.16 |
| Peripheral Vascular Disease, N(%) | 437 (4.2) | 19 (3.7) | 418 (4.2) | 0.584 |
| Hypertension, N(%) | 6377 (61.5) | 308 (60.6) | 6069 (61.6) | 0.673 |
| Diabetes, N(%) | **1876 (18.1)** | **112 (22.0)** | **1764 (17.9)** | **0.018** |
| Chronic Obstructive Pulmonary Disease, N(%) | 865 (8.3) | 36 (7.1) | 829 (8.4) | 0.293 |
| Cancers, N(%) | 1660 (16.0) | 83 (16.3) | 1577 (16.0) | 0.838 |
| Liver Disease, N(%) | 156 (1.5) | 10 (2.0) | 146 (1.5) | 0.379 |
| Plasma glucose on admission, median (IQR) | 6.3 (5.5-7.9) | 6.3 (5.5-7.8) | 6.5 (5.6-8.3) | 0.298 |
| Creatinine on admission, median (IQR) | **0.9 (0.8-1.2)** | **0.9 (0.8-1.2)** | **0.9 (0.8-1.1)** | **0.032** |
| C-reactive protein on admission, median (IQR) | **12.0 (5.0-37.0)** | **12.0 (5.0-38.0)** | **6.0 (3.0-18.0)** | **<0.001** |
| WBC on admission, median (IQR) | **8.8 (7.1-11.3)** | **8.8 (7.2-11.3)** | **8.2 (6.6-10.3)** | **<0.001** |
| Sodium on admission, mean (SD) | 138.24 (4.08) | 138.24 (4.10) | 138.27 (3.45) | 0.894 |
| Cholesterol on admission, mean (SD) | 4.85 (1.31) | 4.84 (1.32) | 5.01 (1.17) | 0.064 |
| Haemoglobin on admission, mean (SD) | - | - | - | - |

SD – standard deviation, IQR – inter-quartile range ; mRS – modified Rankin Scale ; OCSP – Oxfordshire Community Stroke Project; PACS – partial anterior circulation stroke; LACS – lacunar stroke; TACS – total anterior circulation stroke; POCS – posterior circulation stroke; WBC – white blood cell count

Statistically significant results are highlighted in **bold**.

**Supplementary Table 5**. Patient characteristics stratified by whether the data for **creatinine levels** were missing

| Variable | Total | Missing | Non-missing | *P*-value |
| --- | --- | --- | --- | --- |
| Age, mean (SD) | 78.48 (10.90) | 78.51 (10.88) | 77.60 (11.43) | 0.188 |
| Female, N(%) | 5409 (52.2) | 120 (46.5%) | 5289 (52.3%) | 0.065 |
| Pre-stroke mRS, median (IQR) | 0.0 (0.0-1.0) | 0.0 (0.0-1.0) | 0.0 (0.0-1.0) | 0.259 |
| Death, N(%) | **4887 (47.1)** | **89 (34.5)** | **4798 (47.5)** | **<0.001** |
| OCSP classification |  |  |  | **0.004** |
| PACS, N(%) | 3534 (34.1) | 88 (36.7) | 3446 (34.1) |  |
| LACS, N(%) | 2401 (23.2) | 51 (36.7) | 2350 (23.2) |  |
| TACS, N(%) | 1830 (17.7) | 31 (36.7) | 1799 (17.8) |  |
| POCS, N(%) | 1533 (14.8) | 48 (36.7) | 1485 (14.7) |  |
| Coronary Heart Disease, N(%) | **2916 (28.1)** | **47 (18.2)** | **2869 (28.4)** | **<0.001** |
| Peripheral Vascular Disease, N(%) | 437 (4.2) | 11 (4.3) | 426 (4.2) | 0.969 |
| Hypertension, N(%) | **6377 (61.5)** | **137 (53.1)** | **6240 (61.7)** | **0.005** |
| Diabetes, N(%) | 1876 (18.1) | 51 (19.8) | 1825 (18.1) | 0.481 |
| Chronic Obstructive Pulmonary Disease, N(%) | 865 (8.3) | 13 (5.0) | 852 (8.4) | 0.052 |
| Cancers, N(%) | 1660 (16.0) | 35 (13.6) | 1625 (16.1) | 0.278 |
| Liver Disease, N(%) | 156 (1.5) | 3 (1.2) | 153 (1.5) | 0.648 |
| Plasma glucose on admission, median (IQR) | 6.3 (5.5-7.9) | 6.3 (5.5-7.9) | 6.0 (5.5-6.5) | 0.426 |
| Creatinine on admission, median (IQR) | - | - | - | - |
| C-reactive protein on admission, median (IQR) | **12.0 (5.0-37.0)** | **12.0 (5.0-37.0)** | **4.0 (3.0-9.0)** | **<0.001** |
| WBC on admission, median (IQR) | **8.8 (7.1-11.3)** | **8.8 (7.1-11.3)** | **8.1 (6.8-9.9)** | **0.034** |
| Sodium on admission, mean (SD) | **138.24 (4.08)** | **138.24 (4.08)** | **133.40 (2.07)** | **0.008** |
| Cholesterol on admission, mean (SD) | 4.85 (1.31) | 4.85 (1.31) | 5.15 (1.25) | 0.139 |
| Haemoglobin on admission, mean (SD) | 134.43 (19.58) | 134.42 (19.59) | 137.72 (17.58) | 0.341 |

SD – standard deviation, IQR – inter-quartile range ; mRS – modified Rankin Scale ; OCSP – Oxfordshire Community Stroke Project; PACS – partial anterior circulation stroke; LACS – lacunar stroke; TACS – total anterior circulation stroke; POCS – posterior circulation stroke; WBC – white blood cell count

Statistically significant results are highlighted in **bold**.

**Supplementary Table 6**. Patient characteristics stratified by whether the data for **pre-stroke modified Rankin Scale** were missing

| Variable | Total | Missing | Non-missing | *P*-value |
| --- | --- | --- | --- | --- |
| Age, mean (SD) | **78.48 (10.90)** | **78.33 (10.91)** | **81.02 (10.33)** | **<0.001** |
| Female, N(%) | 5409 (52.2) | 333 (54.8%) | 5076 (52.0%) | 0.188 |
| Pre-stroke mRS, median (IQR) | - | - | - | - |
| Death, N(%) | **4887 (47.1)** | **441 (72.5)** | **4446 (45.6)** | **<0.001** |
| OCSP classification |  |  |  | **<0.001** |
| PACS, N(%) | 3534 (34.1) | 163 (34.5) | 3371 (34.5) |  |
| LACS, N(%) | 2401 (23.2) | 86 (34.5) | 2315 (23.7) |  |
| TACS, N(%) | 1830 (17.7) | 158 (34.5) | 1672 (17.1) |  |
| POCS, N(%) | 1533 (14.8) | 73 (34.5) | 1460 (15.0) |  |
| Coronary Heart Disease, N(%) | 2916 (28.1) | 166 (27.3) | 2750 (28.2) | 0.640 |
| Peripheral Vascular Disease, N(%) | 437 (4.2) | 30 (4.9) | 407 (4.2) | 0.364 |
| Hypertension, N(%) | **6377 (61.5)** | **308 (50.7)** | **6069 (62.2)** | **<0.001** |
| Diabetes, N(%) | 1876 (18.1) | 103 (16.9) | 1773 (18.2) | 0.445 |
| Chronic Obstructive Pulmonary Disease, N(%) | 865 (8.3) | 60 (9.9) | 805 (8.2) | 0.161 |
| Cancers, N(%) | 1660 (16.0) | 110 (18.1) | 1550 (15.9) | 0.150 |
| Liver Disease, N(%) | 156 (1.5) | 10 (1.6) | 146 (1.5) | 0.770 |
| Plasma glucose on admission, median (IQR) | **6.3 (5.5-7.9)** | **6.3 (5.5-7.8)** | **6.7 (5.6-8.4)** | **0.022** |
| Creatinine on admission, median (IQR) | **0.9 (0.8-1.2)** | **0.9 (0.8-1.2)** | **1.0 (0.8-1.3)** | **<0.001** |
| C-reactive protein on admission, median (IQR) | **12.0 (5.0-37.0)** | **11.0 (5.0-35.0)** | **28.0 (10.0-77.0)** | **<0.001** |
| WBC on admission, median (IQR) | **8.8 (7.1-11.3)** | **8.8 (7.1-11.2)** | **10.1 (7.6-13.2)** | **<0.001** |
| Sodium on admission, mean (SD) | 138.24 (4.08) | 138.25 (4.04) | 138.13 (4.62) | 0.507 |
| Cholesterol on admission, mean (SD) | **4.85 (1.31)** | **4.86 (1.32)** | **4.63 (1.19)** | **0.007** |
| Haemoglobin on admission, mean (SD) | **134.43 (19.58)** | **134.81 (19.45)** | **127.86 (20.75)** | **<0.001** |

SD – standard deviation, IQR – inter-quartile range ; mRS – modified Rankin Scale ; OCSP – Oxfordshire Community Stroke Project; PACS – partial anterior circulation stroke; LACS – lacunar stroke; TACS – total anterior circulation stroke; POCS – posterior circulation stroke; WBC – white blood cell count

Statistically significant results are highlighted in **bold**.

**Supplementary Table 7**. Patient characteristics stratified by whether the data for **Oxfordshire Community Stroke Project Classification** were missing

| Variable | Total | Missing | Non-missing | *P*-value |
| --- | --- | --- | --- | --- |
| Age, mean (SD) | 78.48 (10.90) | 78.42 (10.89) | 79.04 (10.93) | 0.079 |
| Female, N(%) | 5409 (52.2) | 543 (50.8%) | 4866 (52.3%) | 0.356 |
| Pre-stroke mRS, median (IQR) | **0.0 (0.0-1.0)** | **0.0 (0.0-1.0)** | **0.0 (0.0-2.0)** | **<0.001** |
| Death, N(%) | 4887 (47.1) | 499 (46.7) | 4388 (47.2) | 0.771 |
| OCSP classification | - | - | - | - |
| PACS, N(%) |  |  |  |  |
| LACS, N(%) |  |  |  |  |
| TACS, N(%) |  |  |  |  |
| POCS, N(%) |  |  |  |  |
| Coronary Heart Disease, N(%) | 2916 (28.1) | 289 (27.1) | 2627 (28.3) | 0.411 |
| Peripheral Vascular Disease, N(%) | 437 (4.2) | 40 (3.7) | 397 (4.3) | 0.419 |
| Hypertension, N(%) | 6377 (61.5) | 630 (59.0) | 5747 (61.8) | 0.073 |
| Diabetes, N(%) | 1876 (18.1) | 187 (17.5) | 1689 (18.2) | 0.598 |
| Chronic Obstructive Pulmonary Disease, N(%) | 865 (8.3) | 102 (9.6) | 763 (8.2) | 0.132 |
| Cancers, N(%) | **1660 (16.0)** | **200 (18.7)** | **1460 (15.7)** | **0.011** |
| Liver Disease, N(%) | **156 (1.5)** | **26 (2.4)** | **130 (1.4)** | **0.008** |
| Plasma glucose on admission, median (IQR) | **6.3 (5.5-7.9)** | **6.3 (5.5-7.8)** | **6.5 (5.6-8.2)** | **0.018** |
| Creatinine on admission, median (IQR) | 0.9 (0.8-1.2) | 0.9 (0.8-1.2) | 1.0 (0.8-1.2) | 0.126 |
| C-reactive protein on admission, median (IQR) | 12.0 (5.0-37.0) | 12.0 (5.0-36.0) | 11.0 (4.0-43.5) | 0.958 |
| WBC on admission, median (IQR) | **8.8 (7.1-11.3)** | **8.8 (7.1-11.2)** | **9.2 (7.4-12.1)** | **<0.001** |
| Sodium on admission, mean (SD) | 138.24 (4.08) | 138.25 (4.05) | 138.17 (4.29) | 0.561 |
| Cholesterol on admission, mean (SD) | 4.85 (1.31) | 4.85 (1.31) | 4.80 (1.35) | 0.350 |
| Haemoglobin on admission, mean (SD) | **134.43 (19.58)** | **134.69 (19.42)** | **132.14 (20.82)** | **<0.001** |

SD – standard deviation, IQR – inter-quartile range ; mRS – modified Rankin Scale ; OCSP – Oxfordshire Community Stroke Project; PACS – partial anterior circulation stroke; LACS – lacunar stroke; TACS – total anterior circulation stroke; POCS – posterior circulation stroke; WBC – white blood cell count

Statistically significant results are highlighted in **bold**.

**Supplementary Table 8**. Results of the logistic regression of missing/none missing

| Variable | Odds Ratio (95% Confidence Interval) |
| --- | --- |
| Sex | 0.96 (0.85-1.06) |
| Age | - 1. (1.00-1.01) |
| Atrial Fibrillation | 0.98 (0.87-1.09) |
| Congestive Heart Failure | 1.06 (0.91-1.21) |
| Coronary Heart Disease | 0.89 (0.77-1.01) |
| Cancer | 1.13 (1.00-1.26) |
| Hypertension | 0.81 (0.71-0.91) |
| Chronic Obstructive Pulmonary Disease | 1.05 (0.87-1.23) |
| Liver Disease | 1.63 (1.28-1.99) |
| Peripheral Vascular Disease | 1.02 (0.78-1.27) |

**Supplementary Figures**

**Supplementary Figure 1**. Patient population flowchart.


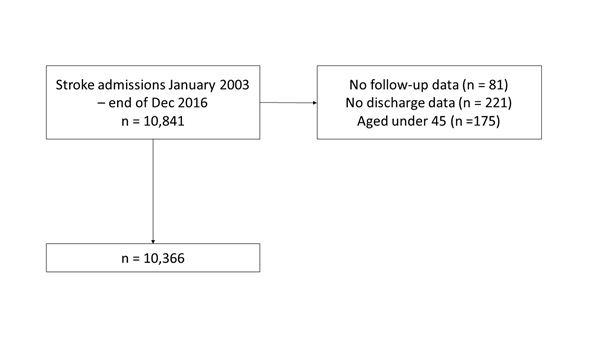


**Supplementary Figure 2.** Results of the multivariable Cox regressions assessing the relationship between blood measurements on admission (haemoglobin, sodium and white blood count levels) and 10-year stroke mortality. Haemoglobin was modelled using restricted cubic splines (RCS) with 5 knots, Sodium RCS with 4 knots and white blood count RCS with 5 knots. Log Hazard ratios and respective 95% confidence intervals are represented by the blue line with grey shadowing. The dotted red line represents the reference line (log HR = 0). The overlaying blue bar chart displays the distribution of each variable in the included cohort.

The multivariable Cox regression included the following predictors: age, sex, Oxfordshire Community Stroke Project, estimated Glomerular Filtration Rate, pre-stroke modified Ranking Scale, co-morbidities (atrial fibrillation, coronary heart disease, congestive heart failure, cancers, hypertension, chronic obstructive pulmonary disease, liver disease and peripheral vascular disease) and blood measurements on admission (haemoglobin, sodium and white blood count levels)


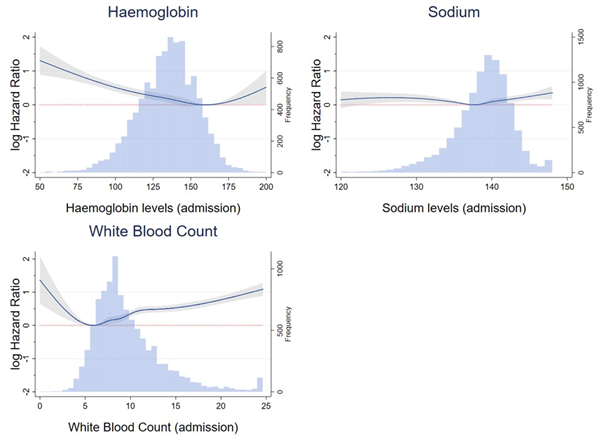


**Supplementary Figure 3.** Distribution of the calculated Score values in the included patient cohort.


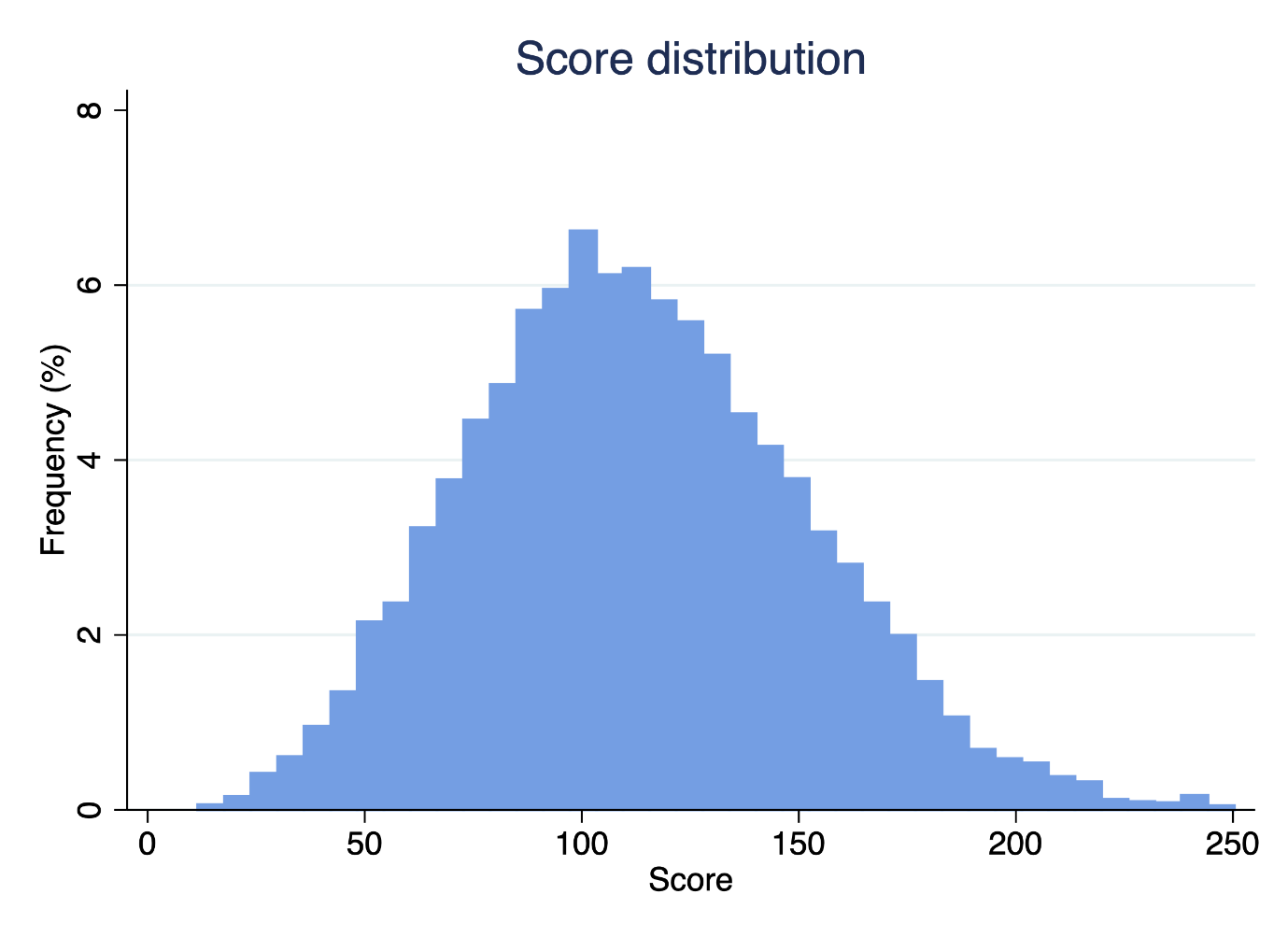


**Supplementary Figure 4.** Observed 10-year survival curves, stratified by score quintiles (Fifth 1: 11.5-79.5, Fifth 2: 79.4,100.9, Fifth 3: 100.9-120.9, Fifth 4:120.9-145.8, Fifth 5: 145.8-250.4)**
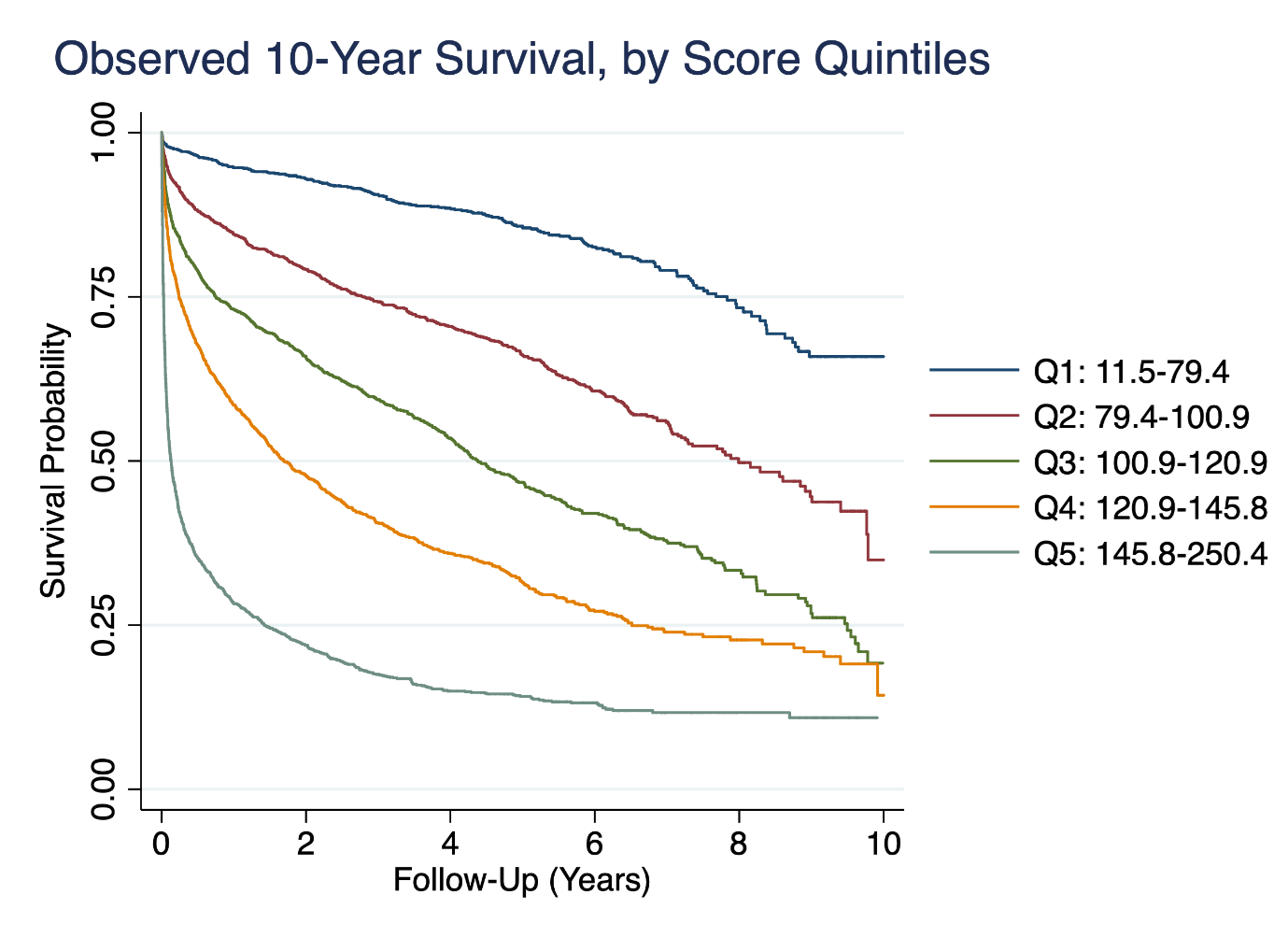
**
